# Supplementary material for: Recombinant Klotho Protein Ameliorates Myocardial Ischemia/Reperfusion Injury by Attenuating Sterile Inflammation
Source: Biomedicines. 2022 Apr 13;10(4):894. doi: 10.3390/biomedicines10040894 (PMC9032004; doi:10.3390/biomedicines10040894)
Supplement: Supplementary file 1 [file biomedicines-10-00894-s001.zip › biomedicines-1627635 - supplement figure.pdf]

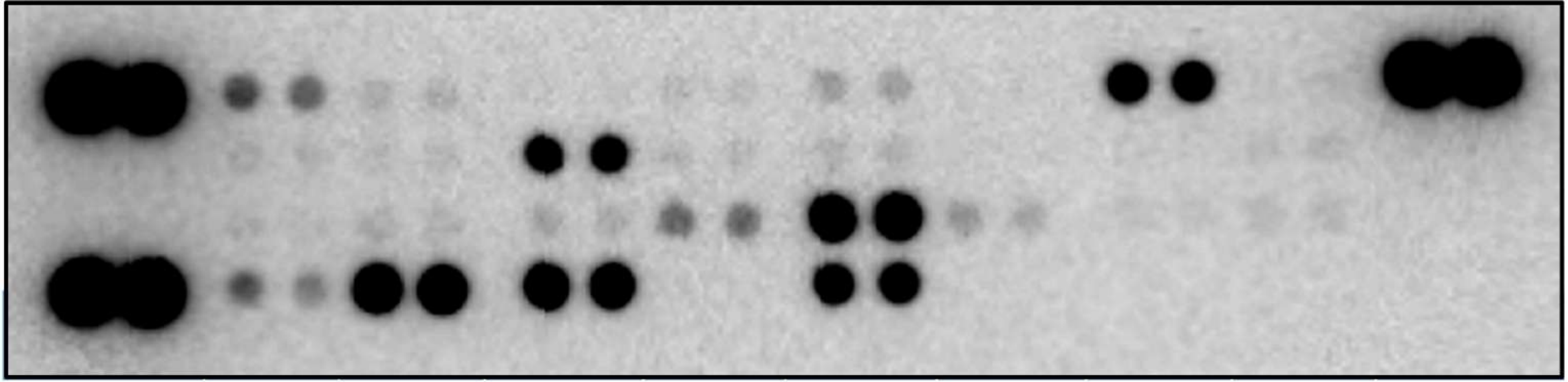

Reference spots	A4 (CINC-1)	A4 (CINC-1)	A8 (CINC-3)	A10 (CNTF)	A12 (Fractalkine)	A14 (GM-CSF)	A16 (sICAM-1)	A18 (IFN-gamma)	Reference spots
	B4 (IL-1alpha)	B6 (IL-1beta)	B8 (IL-1ra)	B10 (IL-2)	B12 (IL-3)	B14 (IL-4)	B16 (IL-6)	B18 (IL-10)	
	C4 (IL-13)	C6 (IL-17)	C8 (IP-10)	C10 (LIX)	C12 (L-Selectin)	C14 (MIG)	C16 (MIP-1alpha)	C18 (MIP-3alpha)	
Reference spots	D4 (RANTES)	D6 (Thymus Chemokine)	D8 (TIMP-1)	D10 (TNF-alpha)	D12 (VEGF)				Negative control
