# Supplementary material for: Recombinant Klotho Protein Ameliorates Myocardial Ischemia/Reperfusion Injury by Attenuating Sterile Inflammation
Source: Biomedicines. 2022 Apr 13;10(4):894. doi: 10.3390/biomedicines10040894 (PMC9032004; doi:10.3390/biomedicines10040894)
Supplement: Supplementary file 1 [file biomedicines-10-00894-s001.zip › biomedicines-1627635 - table.pdf]

biomedicines

MDPI

Table S1. Number (*n*) of Animals in the Present Study

Number (*n*) of Animals	Sham	Sham + Klotho	MI	MI + Klotho
2,3,5-triphenyltetrazolium chloride (TTC) at 4 h after I/R injury in Fig 1				
Representative immunohistochemistry of HMGB1 in Fig 3	5	5	5	5
High mobility group box-1 (HMGB1) in plasma in Fig 3				
2,3,5-TTC at 24 h after I/R injury in Fig 1	-	-	5	5
Intracellular reactive oxygen species (ROS) level in Fig 2	5	5	5	5
Inflammatory cytokine expression in Fig 4				
TUNEL assay in Fig 5	5	5	5	5
cTnT in plasma in Fig 3				
Cytokine array analysis at 24 h in Fig 6	5	5	5	5
Total (*n* = 90)	20	20	25	25

Terminal deoxynucleotidyl transferase (TdT)-mediated dUTP nick end labeling (TUNEL) assay.

Number (*n*) of Animals	MI without rKL (*n*)	MI+ 0.025 µg of rKL/g of Body Mass (*n*)	MI + 0.05 µg of rKL/g of Body Mass (*n*)	MI + 0.075 µg of rKL/g of Body Mass (*n*)
For determination of the optimal dose of rKL for myocardial protection, 2,3,5-TTC at 4 h after I/R injury	3	3	3	3
Total (*n* = 12)	3	3	3	3

Table S2. The Statistical Results for the Optimal Dose of Recombinant Klotho

2,3,5-Triphenyltetrazo-lium Chloride (TTC) (%), Mean ± Standard Deviation (SD)				Overall	Post Hoc Analysis *p*-Value					
MI (1) (*n* = 3)	MI + Klotho 0.025 µg (2) (*n* = 3)	MI+ Klotho 0.05 µg (3) (*n* = 3)	MI+ Klotho 0.075 µg (4) (*n* = −3)	*p*-Value	(1) vs. (2)	(1) vs. (3)	(1) vs. (4)	(2) vs. (3)	(2) vs. (4)	(3) vs. (4)
20.57 ± 3.23	11.3 ± 0.05	5.97 ± 3.42	3.71 ± 0.78	0.001	0.011	0.001	0.001	0.027	0.032	0.730

MI, myocardial infarction.

Table S3. The Statistical Results of the Present Study

	**Mean ± Standard Deviation (SD)**				**Overall**	**Post Hoc Analysis *p*-Value**					
	**Sham (1) (*n* = 5)**	**Sham + Klotho (2) (*n* = 5)**	**MI (3) (*n* = 5)**	**MI + Klotho (4) (*n* = 5)**	***p*-Value**	**(1) vs. (2)**	**(1) vs. (3)**	**(1) vs. (4)**	**(2) vs. (3)**	**(2) vs. (4)**	**(3) vs. (4)**
2,3,5-triphenyltetrazo-lium chloride (TTC)(%)											
4 h TTC (%)	0.0	0.0	15.72 ± 1.65	5.18 ± 1.40	<0.001	1.000	<0.001	<0.001	<0.001	<0.001	<0.001
24h TTC (%)			16.38 ± 6.93	5.22 ± 1.53	0.014						
Reactive oxygen species level (%)											
	0.35 ± 0.23	0.04 ± 0.07	40.70 ± 8.59	8.98 ± 6.84	<0.001	1.000	<0.001	0.246	<0.001	0.211	<0.001
High mobility group box-1 (HMGB1) colonization (%)											
	92.62 ± 2.02	92.25 ± 1.72	18.25 ± 15.04	79.45 ± 5.68	<0.001	1.000	<0.001	0.217	<0.001	0.246	<0.001
HMGB1 Enzyme-linked immunosorbent assay (ELISA) (pg/mL)											
	35.43 ± 3.78	44.11 ± 7.31	104.06 ± 32.18	34.34 ± 10.98	<0.001	1.000	0.001	1.000	0.003	1.000	0.001
Cardiac troponin (cTnT) ELISA in plasma (ng/mL)											
	0.37 ± 0.19	0.70 ± 0.20	3.78 ± 0.99	1.33 ± 0.09	<0.001	1.000	<0.001	0.110	<0.001	0.633	<0.001
Quantification of the expression of inflammatory cytokines											
TNF-α	2.41 ± 0.69	2.28 ± 0.73	18.71 ± 12.28	4.60 ± 0.50	0.004	1.000	0.011	1.000	0.010	1.000	0.031
IL-1β	1.61 ± 0.29	2.69 ± 0.81	73.32 ± 25.21	23.39 ± 3.44	<0.001	1.000	<0.001	0.167	<0.001	0.212	<0.001
IL-6	2.17 ± 0.75	4.27 ± 1.63	3986.57 ± 3176.19	550.19 ± 193.98	0.007	1.000	0.016	1.000	0.016	1.000	0.045
Terminal deoxynucleotidyl transferase (TdT)-mediated dUTP nick end labeling (TUNEL) assay (*n*)											
	1 ± 0.63	1 ± 0.0	93.53 ± 21.86	34.13 ± 9.13	<0.001	1.000	<0.001	<0.001	<0.001	<0.001	<0.001
Proteome Profiler array for cytokines											
CINC-1	12,260.21 ± 2186.33	13,841.58 ± 1341.43	25,498.34 ± 1374.20	15,100.64 ± 1974.13	<0.001	1.000	<0.001	0.869	0.001	1.000	0.002
sICAM-1	25,137.56 ± 2039.55	20,804.38 ± 2429.81	41,183.05 ± 4705.14	26,346.87 ± 3858.54	0.002	1.000	0.010	1.000	0.002	0.869	0.015
LIX	11,337.85 ± 1226.16	12,200.12 ± 1239.62	20,287.01 ± 3009.43	13,248.30 ± 1668.39	0.006	1.000	0.010	1.000	0.018	1.000	0.039
L-Selectin	17,683.36 ± 2987.18	19,458.25 ± 2229.08	51,362.90 ± 3984.12	31,617.62 ± 3600.89	<0.001	1.000	<0.001	0.016	<0.001	0.035	0.002

MI, myocardial infarction; TNF, tumor necrosis factor; IL, interleukine; CINC-1, Cytokine-induced neutrophil chemoattractant 1; sICAM-1/CD54, soluble intracellular adhesion molecule-1; LIX, Chemokine (C-X-C motif) ligand 5 (CXCL5).
